# Designing a comprehensive Non-Communicable Diseases (NCD) programme for hypertension and diabetes at primary health care level: evidence and experience from urban Karnataka, South India

**DOI:** 10.1186/s12889-019-6735-z

**Published:** 2019-04-16

**Authors:** Krishnamurthy Jayanna, N. Swaroop, Arin Kar, Satyanarayana Ramanaik, Manoj Kumar Pati, Ashwini Pujar, Prathibha Rai, Suresh Chitrapu, Gururaj Patil, Preeti Aggarwal, Shivla Saksena, Hemanth Madegowda, S. Rekha, H. L. Mohan

**Affiliations:** 1 Karnataka Health Promotion Trust, Bangalore, India; 2 Centre for Global Public Health, University of Manitoba, Winnipeg, Canada; 3 Landmark group, Bangalore, India; 4 Department of Health and Family Welfare, Government of Karnataka, Bangalore, Karnataka India; 5 IT Park, Rajajinagar Industrial Area Behind KSSIDC Admin office, 5th floor, 1-4, Rajajinagar, Bengaluru, Karnataka 560044 India

**Keywords:** Integrated health service delivery, Continuum of care, Urban health, Primary care, Non Communicable diseases programs, hypertension, diabetes

## Abstract

**Background:**

India accounts for more than two-third of mortality due to non-communicable diseases (NCDs) in south-east Asia. The burden is high in Karnataka, one of the largest states in southern India. There is a need for integration of disease prevention, health promotion, treatment and care within the national program at primary level. A public-private partnership initiative explored evidence gaps to inform a health system based, integrated NCD programme across care continuum with a focus on hypertension and diabetes.

**Methods:**

The study was conducted during 2017–18 in urban parts of Mysore city, covering a population of 58,000. Mixed methods were used in the study; a population-based screening to estimate denominators for those with disease and at risk; cross-sectional surveys to understand distribution of risk factors, treatment adherence and out of pocket expenses; facility audits to assess readiness of public and private facilities; in-depth interviews and focus group discussions to understand practices, myths and perceptions in the community. Chi-square tests were used to test differences between the groups. Framework analysis approach was used for qualitative analysis.

**Results:**

Twelve and 19% of the adult population had raised blood sugar and blood pressure, respectively, which increased with age, to 32 and 44% for over 50 years. 11% reported tobacco consumption; 5.5%, high alcohol consumption; 40%, inadequate physical activity and 81%, inappropriate diet consumption. These correlated strongly with elderly age and poor education. The public facilities lacked diagnostics and specialist services; care in the private sector was expensive. Qualitative data revealed fears and cultural myths that affected treatment adherence. The results informed intervention design across the NCD care continuum.

**Conclusions:**

The study provides tools and methodology to gather evidence in designing comprehensive NCD programmes in low and middle income settings. The study also provides important insights into public-private partnership driving effective NCD care at primary care level.

**Electronic supplementary material:**

The online version of this article (10.1186/s12889-019-6735-z) contains supplementary material, which is available to authorized users.

## Background

World Health Organization (WHO) estimates that more than a third of 40 million deaths occurring due to Non-Communicable Diseases (NCDs) globally, are premature deaths. Over 80% of them occur in low- and middle-income countries (LMIC) [1]. With around 5.87 million deaths annually, India shares more than two-third of the mortality due to NCDs in the South-East Asia Region^1^ [2]. The probability of an Indian, in the age group of 30–70 years, dying at present from one of the four NCDs i.e. cardiovascular diseases (CVD), diabetes, cancer, and chronic respiratory disease is 26% [3]. Burden of NCDs in India is expected to worsen in the future; diabetics will increase from 40.9 million to 69.9 million by 2025 and obesity will affect 52.1 million by 2030 [4, 5]. CVD will be the leading cause of death contributing to 29% of all deaths by 2030. Clearly, there is a sense of urgency to address a two-fold challenge i.e. to manage the large cohort of NCD cases effectively and to prevent new additions into this cohort. The sustainable development goal (SDG) 3.4 aims to reduce the premature mortality due to NCDs by a third [6] and absence of timely and effective intervention at this juncture may leave the hope of achieving the SDG unrealized.

The burden of NCDs is high in the state of Karnataka, one of the largest states in southern India. As per recent estimates, four major NCDs constitutes around 25% of all disease burden among 15–39 aged population in the state; this burden reaches more than 70% among people aged more than 40 years [7]. A study done in coastal Karnataka in 2006–07 showed the prevalence of hypertension among people over 30 years at 43.3% and out of them, only half knew that they had hypertension, and 20.2% were newly detected during the study. The prevalence of type 2 diabetes ranges from 3.77 to 16% [8, 9].

The Ministry of Health and Family Welfare of Government of India launched the National Programme in 2008 with the objectives to prevent and control common NCDs through behaviour and life style modification, early diagnosis and management of common NCDs such as hypertension and diabetes, building capacity at various levels of health care. To strengthen implementation, a national NCD cell was constituted to develop standard operating procedures, training modules, operational guidelines, quality benchmarks, monitoring tools and reporting formats [10]. National Health Mission (NHM) provides an overarching umbrella, subsuming the existing NCD control programmes in both urban and rural areas. The efforts have not been effective on the ground due to implementation level challenges. Health systems constraints in relation to human resources, poor training quality, and poor awareness within community have been highlighted [11]. Experts are also critical about the lack of strategic focus on specific disease conditions within the larger NCD programs [12].

In this context, there is a need to strengthen implementation on the ground. The need to implement the programme comprehensively, through integration of disease prevention, health promotion, treatment and care at the primary level, is well envisaged in policies and guidelines. But there is a paucity of evidence as to how best to integrate them on the ground [10]. It is particularly important, in the context of fragmented urban health systems, to understand as to how to design, implement, monitor and evaluate NCD interventions across its care continuum [13–16]. To address this knowledge gap, a pilot implementation research initiative was undertaken during 2017–18 in urban city of Mysore in Karnataka. The consortia had a mix of public and private partners i.e. Government of Karnataka, Landmark group and Karnataka Health Promotion Trust (KHPT). The objectives of the implementation research were to undertake an in-depth situation analysis through a mixed methods approach, and design effective health systems based interventions through an iterative formative research process. The project focused its efforts on two common NCDs, hypertension and diabetes. In this paper, we present findings and insights from the research that informed the design of the intervention model. The paper also presents the model that will be further evaluated through a formative research, concurrent monitoring and impact evaluation in the next phase.

## Methods

### Study design and framework

We used mixed methods research design to identify gaps and design interventions for Hypertension and Diabetes across the care continuum using an established continuum of care framework [17]. Through a series of workshops, the project teams identified the information gaps and the plans for situation analysis (Table 1).

**Table 1:** Information gaps for designing NCD interventions

#	Information pre-requisite	Utility of information	Level of action (intervention)
1	Population burden of diabetes and hypertension, their risk factors	To plan adequate resources and infrastructure for screening and management, prevention, promotion	Facilities and frontline health workers in the community
2	Facility readiness to offer NCD care	To plan interventions to strengthen the availability and quality of NCD care	Health systems and Facility level
3	Community characteristics: Lifestyles, treatment adherence, out of pocket expenses.	To plan adequate prevention and health promotion measures	Facilities and frontline health workers in the community
4	Community characteristics: myths, beliefs and practices around hypertension and diabetes that affect health seeking and compliance	To plan educational and behaviour change initiatives	Individual, family and community level

### Study setting and population

The study was carried out in Kumbarakoppalu urban primary health center (UPHC) area in Mysore, a city located in South of Karnataka. Mysore is known for its tradition and culture, with tourism and agriculture driving the economy. The city has population of 990,900 (50.2% men and 49.8% women), a sex ratio of 993 and child sex ratio of 955. The city has a literacy rate of 87.5%; 73% of the population belongs to Hindu religion, and 17% represent the disadvantaged section, referred to as scheduled caste and tribes (SC/ST).^2^

### Study phases, sample, and instruments

The study was implemented between September 2017 and March 2018 in different phases; phase 1: Population-wide screening to detect prevalence of hypertension and diabetes in UPHC area; phase 2: Sample survey to understand distribution of risk factors for hypertension and diabetes, between those who had disease and those who did not; this was followed by survey of all clients (who had hypertension and diabetes) to understand their out of pocket expenses and treatment adherence. Facility audits were undertaken to assess readiness to provide care for hypertension and diabetes; phase 3: Qualitative research (focus group discussions(FGDs), and in-depth interviews (IDIs)) were conducted to understand client experiences and perceptions on NCD care, management, and lifestyle changes.

Phase 1 study targeted all adults aged 18 years and above in the UPHC area. An initial listing followed by adjusting for inaccessible households using census data helped to estimate the population denominator of 37,943. The screening tool had two sections. 1) Household section, that captured address and age-gender composition of members, 2) NCD section captured values of systolic/ diastolic blood pressure as measured by a digital sphygmomanometer, blood glucose levels as measured by point of care (POC) random blood sugar tests (RBS) and anthropometric tests (height, weight) for calculation of body mass index (BMI). An additional file describes specifications of diagnostics used in the study (ref: Additional file 1).

Phase 2 study of risk factors was undertaken on a random sample of 1470 individuals out of the study population. The sample was estimated using the NCD prevalence from phase 1 study, adjusted with 80% power, 95% confidence interval, a design effect of 2 and 25% of non-response. The households were first selected by a systematic random sampling after which adults were selected randomly using the KISH grid.^3^ The WHO’s STEP’s questionnaire was used to assess risk factors in the sample population. The questionnaire captured socio-demographic details in addition to four modifiable risk factors i.e. alcohol consumption, tobacco consumption, inadequate physical activity and inappropriate diet (ref: Additional file 2). All those who had disease were followed with another questionnaire to understand out of pocket expenses and treatment adherence (ref: Additional file 3). Facility audits were done in 11 out of 12 health facilities, that included 4 public facilities and 7 private facilities. 1 private facility refused to share information with the study team because of apprehensions of possible misuse of data by the government. The audit tool (ref: Additional file 4) captured data about human resources, NCD related medicines and diagnostics and documentation systems. We used the point of care diagnostics and W.H.O’s STEP’s questionnaire that were already validated and adapted for local context. The facility audit tool was pre-tested prior to its use.

The phase 3 study was undertaken after completion of the quantitative study with an objective of getting deeper insights into treatment access, adherence, practices among the subjects that had one of the two NCDs. 30 subjects including 11 men and 19 women were purposively selected for in-depth interviews and focus group discussions. About 20 respondents were above 45 years and the remaining, between 35 and 45 years of age. In terms of education, 16 were illiterate, and among the remaining, five, seven and two subjects had completed primary, high school and college education, respectively. Similarly, in terms of occupation, majority (18) were daily wage labourers and the remaining were house wives (5), priests (2), self-employed (2), driver (1) and retired Government employee (2). We conducted two focused group discussions with 20 participants (9 men and 11 women) and 10 in-depth interviews (2 men and 8 women). A semi-structured interview guide was used (ref: Additional file 5), that consisted several broad themes for the probing: general health-seeking behaviour, knowledge & practices about hypertension and diabetes, reasons for not accessing treatment, treatment adherence and life style modifications challenges. The interview tool was field tested to refine the structure and phrasing for better clarity. Table 2 summarizes different phases of study and the tools used.

**Table 2:** Study phases and tools

PHASE	Method	Category (sample)	Tools	Key variables captured
Phase 1	Quantitative	Population based screening (37,943, > 18 years)	Screening tool and diagnostics	Blood pressure, blood sugar, anthropometric tests
Phase 2a	Quantitative	Sample survey (1470)	W.H.O’s STEP questionnaire	Risk factors (tobacco, alcohol, physical activity and diets) related information
Phase 2b	Quantitative	Survey of all those who had disease (hypertension and diabetes) (157)	Interview Questionnaire	Out of pocket expenses, treatment adherence related details
Phase 2c	Quantitative	Facility audits (12)	Audit tool	Human resources, medicines, diagnostics, documentation systems related details
Phase 3	Qualitative	Focus group discussions (20) and in- depth interviews (10)	FGD guide and interview guide	Knowledge & practices about hypertension and diabetes, treatment adherence and life style modification challenges

### Data collection

19 field investigators from nursing background were supported by a study coordinator for data collection. They received a 3-day training on various topics ranging from NCD epidemiology, definitions, disease signs and symptoms, diagnostics, current treatment standards, community and health systems context, and use of tools. The training included both theoretical lectures, practice sessions in the training room and in the field. The training was done by a team of public health, clinical, and research experts from KHPT. The quantitative data was captured using a computer-assisted personal interviewing (CAPI) based mobile application.

The qualitative data was collected by three researchers (2 males and 1 female) who were well versed with qualitative interview techniques, and had familiarity with local culture and context. The initial pre-testing helped to validate the tools prior to data collection. FGDs were conducted in UPHC premises and the IDIs were carried out in either the homes of respondents or at the health facilities. All interviews were audio recorded.

Data collection was monitored on the field to prevent non-sampling errors. Quality assurance was achieved at various stages through standardization of interview methods, and onsite and offsite reviews by supervisors to monitor correctness and completeness of data. The mobile application (CAPI) had inbuilt validations to prevent errors in coding and completeness.

### Data analysis

The quantitative data were analysed using STATA SE 14. Screening data was analysed to provide estimates for prevalence of high blood sugar and high blood pressure. The sample survey data was analysed by various socio-demographic correlates to understand the characteristics of individuals with disease and risk factors. The difference between the groups were tested using Chi-square tests.

The qualitative data collection and analysis was iterative and occurred simultaneously. The interviews and focused group discussions were translated into English and analysed using framework method [18]. All interviews were coded manually and a matrix with detailed description of various themes was developed.

## Results

### Prevalence of high blood sugar and high blood pressure (Tables 3 and 4)

93% of the estimated population (32596) was contacted by the field investigators. The study population averaged 39 years of age and had more women (52.13%). More than 99% of them consented for screening. Records were checked for confirmation of those who reported as already having disease. 12% of the screened population showed raised blood sugar level; half of them were diagnosed for the first time. Similarly, 19% of the population had high blood pressure and a 2/3rd of them were detected for the first time. Elderly age (above 50 years) showed a highly significant correlation for high blood sugar and high blood pressure across all three categories. More women were detected with raised blood sugar for the first time than men during screening, while it was otherwise for raised blood pressure. No gender difference was observed for previously detected high blood sugar (confirmed diabetes), but more women reported previously diagnosed hypertension than men and these differences were highly statistically significant.

**Table 3:** Prevalence of high blood sugar

Category	Total		Gender break up					Age break up						
			Men		Women		*P*	18 to 34 years		35 to 49 years		50 y and above		*P*
	n	%	n	%	n	%		n	%	N	%	n	%	
Total individuals assessed	32,355	99.26	15,532	48.00	16,823	52.00		14,662	45.32	10,422	32.2	7271	22.47	
High blood sugar detected through screening test^a^ (newly detected)	1916	5.92	870	5.60	1046	6.22	0.02	296	2.02	743	7.13	877	12.06	< 0.001
High blood sugar previously detected (confirmed diabetics)	2034	6.24	984	6.31	1050	6.18	0.63	47	0.32	551	5.26	1436	19.67	< 0.001
Total	3950	12.12	1854	11.88	2096	12.33	0.21	343	2.31	1294	12.36	2313	31.68	< 0.001

^a^Note: Screening test used random blood sugar (RBS) value of more than 140 mg/dl as high blood sugar as per national guidelines

**Table 4:** Prevalence of raised blood pressure

Category	Total		Gender break up					Age break up						
			Men		Women		*P*	18 to 34 y		35 to 49 y		50 y and above		*P*
	No	%	N	%	n	%		n	%	N	%	n	%	
Total individuals assessed	32,568	99.91	15,588	47.86	16,980	52.14		14,819	45.50	10,459	32.11	7290	22.38	
High blood pressure detected through screening test^a^ (newly detected)	4207	12.92	2168	13.91	2039	12.01	< 0.001	865	5.84	1702	16.3	1640	22.50	< 0.001
High blood pressure previously detected (confirmed hypertensives)	2126	6.52	865	5.54	1261	7.42	< 0.001	36	0.24	508	4.85	1582	21.67	< 0.001
Total	6333	19.43	3033	19.44	3300	19.42	0.95	901	6.08	2210	21.1	3222	44.14	< 0.001

^a^Note: Screening test used systolic blood pressure above 140 and diastolic blood pressure above 90 mmHg as per national guidelines

### Population distribution of risk factors (Table 5)

One thousand three hundred nineteen individuals responded to the survey at 89.7% response rate. Their mean age was 40 years; 53.37% were women; 12% were from scheduled tribe & caste groups; 18% never went to school, and 74% were married. Further, 11% of the interviewed individuals reported tobacco consumption; 5.5%, high alcohol consumption; 40%, inadequate physical activity and 81%, inappropriate diet consumption. Tobacco use was significantly higher among men, elderly, and those with fewer years of education; high alcohol use among men and elderly; inadequate physical activity with women, elderly and fewer years of education; inappropriate diet was high across all categories of age, gender, education, and caste groups.

**Table 5:** Distribution of NCD risk factors in the study area

		Tobacco use			Alcohol use^a^				Physical activity^b^			At least 2 or more times Vegetables or fruits in a day^c^		
	N	Yes (%)	No (%)	*p*	High (%)	Low (%)	None (%)	*P*	Adequate (%)	Inadequate (%)	*p*	Yes (%)	No (%)	*p*
Total	1319	10.84	89.16		5.53	4.17	90.30		60.05	39.95		18.65	81.35	
Age														
*< 35 years*	*524*	6.30	93.70	<.001	3.63	2.29	94.08	<.001	64.50	35.50	<.001	79.77	20.23	0.489
*35–49*	*475*	11.58	88.42		5.68	4.42	89.89		62.74	37.26		82.32	17.68	
*50 and above*	*320*	17.19	82.81		8.44	6.88	84.69		48.75	51.25		82.50	17.50	
Education														
*< 5 years*	*289*	17.99	82.01	<.001	6.92	3.46	89.62	0.065	47.40	52.60	<.001	84.78	15.22	0.205
*5–11 years*	*553*	11.97	88.43		6.87	3.98	89.15		61.66	38.34		81.01	18.99	
*12 and above*	*477*	5.66	94.34		3.14	4.82	92.03		65.83	34.17		79.66	20.34	
Gender														
*Male*	*615*	21.95	78.05	<.001	11.54	8.78	79.67	<.001	72.20	27.80	<.001	78.86	21.14	0.03
*Female*	*704*	1.14	98.86		0.28	0.14	99.57		49.43	50.57		83.52	16.48	
Caste														
*scheduled caste and tribe (SC/ST)*	169	14.20	85.80	0.132	6.51	5.92	87.57	0.385	61.54	38.46	0.671	81.66	18.34	0.913
*Others*	*1150*	10.35	89.65		5.39	3.91	90.70		59.83	40.17		81.30	18.70	

^a^High consumption is considered as consumption of 90 ml or more during one event of drinking

^b^Less than at least 75 min of vigorous or 150 min of moderate physical activity per week is considered to be inadequate as per WHO STEPS guideline

^c^Appropriate diet is considered as at least 5 or more times consumed fruits or vegetables/tubers/green leafy vegetables (WHO STEPs Guidelines). Since we did not find anyone, we used at-least 2 serves of fruits/ vegetables for analysis

### Treatment adherence and out of pocket expenses by clients (Table 6)

Out of the surveyed population, 157 individuals (11.9%) had one or both NCDs. The mean duration of illness reported, was lowest for hypertension and highest for coexisting NCDs (hypertension and diabetes). They reported an average time interval of one month between diagnosis and seeking care at a nearby facility. More than 94% reported that they visited clinics regularly for follow up check-ups and to collect medicines. 92% had medicines at the time of the survey and 78% reported as consuming them regularly. However, on actual assessments of RBS and BP, less than 54 and 27% respectively, demonstrated optimal control of the disease; this was poorest among those who had co-existing NCDs (14%). The average annual expenditure was 42 USD for Diabetes care and 25 USD for Hypertensive care; over 50% of expenditure was on medications. The expenditure in private was five times higher than in public facilities (51 USD Vs 9 USD).

**Table 6:** Health seeking behaviour, treatment adherence and out of pocket expenses among people with diabetes and hypertension

Section 1:Health seeking behaviour and treatment adherence				
Parameter	Hypertension (*N* = 64)	Diabetes (*N* = 49)	Co-existing hypertension and diabetes (*N* = 44)	
Average duration of NCD (in years)#	4.34 y	6.19 y	8.07 y	
Clients reported visiting facility at least once in last 12 months (%)	93.55	95.83	95.45	
Number of visits in a year (Average)	4.56	4.44	5.41	
Clients that had medicines at the time of interview (%)	92.06	97.92	97.73	
Clients reported as consuming medicines regularly (%)	77.78	79.59	86.36	
Clients that demonstrated NCD control as per investigations on the day of the interview (%)^a^	26.85	53.76	-NA-	
Section 2: Average annual expenditure on NCD (USD) by disease type and facility type				
Category	Diabetes	Hypertension	Public sector	Private sector
Medicines	21.42	13.85	4.43	26.58
Investigations	12.00	5.72	2.22	14.03
Consultation	8.12	5.91	2.40	10.52
Cumulative	42.54	25.48	8.68	50.95

^a^CD control is good if blood pressure < 140/90 mm of Hg for Hypertension and blood sugar < 200 mg/dl for Diabetes

### Facility readiness (Table 7)

Four public and Seven private hospitals in the vicinity of the population were audited. Five of them (2 public and 3 private) facilities offered primary care whereas the rest offered specialist services. Number of facilities that had at-least one staff, or one dose/ unit of drugs and diagnostics are shown in the table. Public primary care facilities were better staffed than private clinics with respect to pharmacist, lab technicians and staff nurse positions. Specialist positions were filled up in both sectors. NCD counsellor, an important player at primary care level was missing in both sectors. Both types of facilities showed availability of equipment, basic diagnostics and drugs. However, HbA1C (Haemoglobin A1c: Glycated Haemoglobin) test that helps to understand the long-term control of blood glucose, lipid tests and electrocardiogram (ECG) were mostly available in higher facilities. Advanced diagnostics like ECHO (echocardiogram), retinal function tests and kidney function tests to detect complications in the heart, eyes and kidneys, respectively were poorly available across the facilities. NCD related records were better maintained in public facilities.

**Table 7:** No of facilities that had availability of staff, drugs, equipment and supplies

Section 1: Staff availability					
#	Staff Designation	Public (*n* = 4)		Private (*n* = 7)	
		UPHC (*n* = 2)	Higher hospitals (n = 2)	Primary Clinics (*n* = 3)	Higher hospitals (*n* = 4)
1	Endocrinologist/Diabetologist	0	2	0	4
2	Cardiologist	0	1	0	1
3	MBBS Medical Officer	2	2	2	2
4	Alternative Medicine Medical Officer	0	0	1	1
5	Pharmacist	2	2	0	2
6	Laboratory Technician	2	2	0	3
7	NCD Counsellor	0	2	0	1
8	Junior Health Assistant/Staff Nurse	2	2	0	3
9	Data Entry Operator	1	2	0	1
10	Office Assistant	2	2	1	3
Section 2: Availability of diagnostics, treatments, equipment and records					
#		Public (*n* = 4)		Private (*n* = 7)	
	Availability of basic equipment	UPHC (*n* = 2)	Hospitals (*n* = 2)	Primary Clinics (*n* = 3)	Hospitals (*n* = 4)
1	Adult Weighing Scale	2	2	3	4
2	Blood Pressure Apparatus	2	2	3	4
3	Stethoscope	2	2	3	4
4	Stature Meter *(for Height measurement)*	2	2	3	4
	Availability of diagnostics				
5	Blood glucose- FBS, PPBS	2	2	3	4
6	HbA1C	0	1	0	3
7	Lipid Profile	0	1	0	3
8	Electrocardiogram (ECG)	0	2	0	2
9	Echocardiograph (ECHO)	0	0	0	1
10	Retinal examination	0	0	0	0
11	Kidney function tests	0	1	0	2
	Availability of medicines				
12	Oral anti- diabetics	2	2	2	4
13	Oral anti-hypertensives	2	2	2	4
14	Insulin- Injection/ Premix	1	2	2	3
15	Anti-dyslipidaemia drugs	1	2	2	4
16	Documentation				
17	NCD screening register/follow up register	2	1	0	1
18	Laboratory register	2	1	0	1

### NCD related concerns, challenges, and perspectives

Qualitative findings revealed deeper insights into beliefs, practice, treatment access and adherence of those who had hypertension or diabetes. Most of them did not have a clear idea about the cause of disease. Few said it is related to stress and others identified the disease with symptoms such as blurring of vision, tiredness, short temperedness, difficulty in performing physical activities, swelling in the legs and sweating. Most expressed apprehension about their disease and one said,

“*I am never tensed, despite that, I got it why?” I know It occurs genetically- “but none of our ancestors had this…why I got this?” (Somashaker, male, age 35–49)*

Few expressed their anxieties about dietary recommendations and challenges in addressing them due to social circumstances. One male aged 60 years referred that the NCDs have snatched away their regular foods and now they are not supposed to eat non-vegetarian foods. Another woman reported,

*“When we go to others house, they offer tea and coffee with sugar unknowingly… then, we are not able to avoid their hospitality”* (Nagarathna, female, age 50 and above)

Participants have reported many reasons for poor adherence to treatment. Culturally, people take medicines only when they are sick and hence reported discomfort in consuming pills on a daily basis. One respondent expressed fear that the daily consumption of medications would lead to the kidney dis-functioning in the long run. Other reasons included fear about injectable drugs, dislike for the smell of pills, forgetfulness because of a busy schedule, lack of confidence on the effectiveness of treatments. One female participant, aged over 50 years remarked that she preferred to pass away silently instead of letting the world know that she suffered a disease.

With regards to the lifestyle modification, many respondents expressed that their regular work involves physical activity and hence they don’t require additional exercise. One of them said, “*I am doing a carpentry job, I climb 6-7 floors a day and I don’t think I need any additional exercise” (Suresh, male, age 35–49).* Many women and men said that they never had the practice of walking or exercising in the mornings and now feel awkward to do so in front of other community members.

The narratives indicated that alcohol consumption is common among men who cited reasons such as physical tiredness and mental stress. Few considered that taking medication after consuming alcohol will lead to side effects.

*“I generally get tired by doing physical work and therefore, sometimes I drink alcohol. I am scared that alcohol and tablets may lead to some reactions. So, when I drink alcohol, I don’t consume my BP and sugar tablets” (Samaresh, male, age 35–49)*

Female respondents shared their apprehension about taking medication along with their habit of chewing *gutka* (a form of chewable tobacco).

*“I have been chewing gutka since many years and I haven’t had any problems. But now, I was diagnosed with sugar (diabetes) and thyroid problems and started taking treatment for it…If I don’t chew the tobacco, I experience headache and giddiness and I am not able to stop it now…now I am worried about continuing treatment along with my habit of chewing gutka” (Sannamma, female, age 35–49)*

Most of the clients accessed private hospitals and stated reasons such as easy accessibility and availability of doctors, in-spite of paying more for services. Factors such as long waits, lack of attention by doctors and lack of availability of medicines discouraged use of the public facility. The daily wage laborers reported challenges in visiting the public facilities during daytime.

## Discussion

The study presents the scenario of hypertension and diabetes, associated risk factors, health system readiness, prevailing myths and beliefs in the community influencing the health-seeking behaviour. The findings highlight the gaps across the disease care continuum, in a very comprehensive manner at the population level.

Previous studies have reported the regional prevalence for hypertension between 26 and 30% with a higher rate in urban areas at 31% [19, 20]. The current study has reported a prevalence of 19%. Though this is on the lower side when compared to the southern Indian region, it is still higher than the state aggregate that ranges between 9 and 16%. Wide regional variations are reported in the past that are attributable to variations in dietary practices, life styles and urbanization. Similarly, other studies have reported the prevalence of diabetes at around 9.5%. Particularly in urban southern India, it is on the higher side at 11.2%, which is consistent with the current study findings [21].

Furthermore, as per the recent national family health survey report (NFHS, round 4, 2015–16) for the state of Karnataka, specifically in urban areas, the prevalence of raised blood sugar was 7.8% (women) and 9.4% (men), and raised blood pressure, was 9.8% (women) and 16.3% (men). Thus, when compared to NFHS results for the state, the current study estimates a higher prevalence, making the study site one of the most vulnerable pockets in the state of Karnataka. Unlike the state-wide pattern that showed a marked gender difference for both NCDs, the current study population showed only a marginal difference. However, there were some differences among first time detected and previously diagnosed cases. Among the first timers, there were more women than men for high blood sugar, and more men than women for high blood pressure. Also, more women reported as already been diagnosed as hypertensives. This is in contrast to the NFHS findings where more men were detected with high blood pressure and blood sugar. Few other studies have reported similar variations [22, 23]. However, the rise in prevalence with age that was reported by the current study is consistent with the pattern reported in the rest of the state [24].

The basis of NCD prevention is the mitigation of the most common risk factors. W.H.O lays thrust on surveillance of major modifiable risk factors i.e. tobacco consumption, high alcohol consumption, unhealthy diet and insufficient physical activity [25]. The prevalence of risk factors, especially high alcohol consumption and inadequate physical inactivity was very high in the study population when compared to national aggregates (5.5% Vs 1.6%; 40% Vs 13.4%) [19]. Though the study area is recognised as an urban setting, some parts are going through a transition from rural to urban setting. Many people aged above 50 years are not used to outdoor physical activity as they consider it to be culturally inappropriate as reported in the qualitative study. Few men are engaged in physically demanding jobs such as masonry, carpentry etc. and have reported consumption of alcohol to relieve the pain and stress. The correlation with elderly age, gender, and poor education was consistent with the findings reported in other studies in the region [21, 26].

The population reported healthy behaviours in relation to accessing health care i.e. regular clinic visits, consumption of medicines, etc., yet the control of hypertension and diabetes was suboptimal. These findings raise questions about compliance with prescribed treatment, adoption of healthy lifestyles and quality of treatment, as reflected in the qualitative findings and facility audits. Other studies have reported similar findings both in India [26, 27] and in countries of Asia and Africa [28, 29]. Another important finding revealed by the study is the poor readiness of health systems to offer care for the two NCDs. Particularly the primary care in the private sector, and specialist care in the public sector needs attention considering the fact that people are accessing care predominantly in the private sector while spending high out of pocket expenses. Similar observations are reported by other studies locally [30, 31].

The qualitative study sheds light on possible factors influencing poor adherence and hence control of hypertension or diabetes in Mysore city. Misconceptions about the causes of illness alongside anxiety and stigma about the disease is widely prevalent. Non-acceptance of disease status, fears about medications and side effects, cultural beliefs, social norms and challenges faced with health systems are affecting adoption of NCD friendly lifestyles. Studies in Ghana and India have reported similar factors associated with poor adherence [19, 24].

Thus the study provided valuable information at population level that includes, 1) prevalence of high blood pressure and blood sugar, and distribution of risk factors, 2) health system readiness, 3) health-seeking behaviors, myths, and perceptions affecting adherence. Evidence suggests that for high impact of chronic disease control programmes, action has to be directed at 3 levels, i.e. population-wide policies, health services, and community activities, with due emphasis on community involvement and responsive health services [32–34]. Decentralized health services, local government, and public health capacitation in designing, implementation, monitoring and evaluation of interventions, are emphasized [32]. Evidence also points toward 4 important facets for NCD control, i.e. quality improvement, health systems (essential diagnostics, medicines, etc.), decision support (adherence to medications, to follow up, communication with specialists), human resources (staff training, dedicated NCD staff) [33]. Emerging global evidence and insights were adapted to the local study context to identify interventions through a series of workshops and consultations with stakeholders, district officials, providers and community members.

**Fig. 1:**
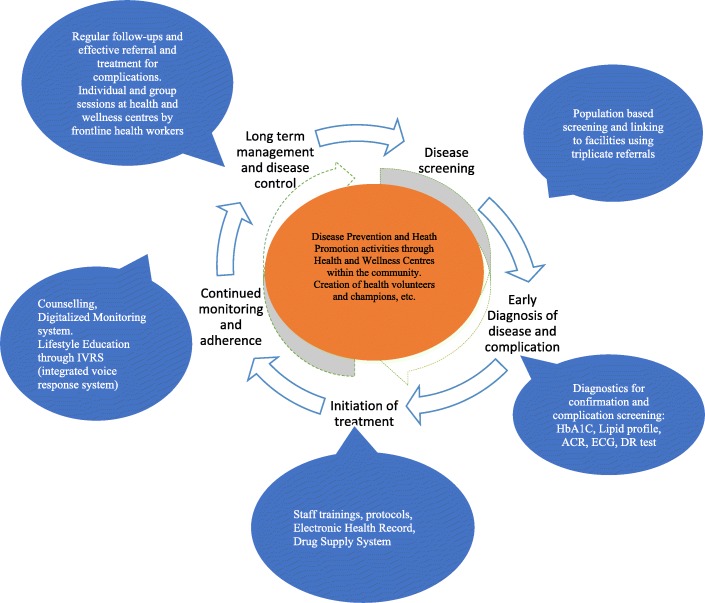
Interventions proposed through NCD continuum of care framework

Interventions were designed at different levels: community, facilities and health systems. Hiring a dedicated NCD counsellor to strengthen counselling in the PHC, upgrading the standards through refresher training of staff and provision of job-aids, digitalizing client health records to facilitate smooth retrieval of information, access to advanced diagnostics at primary level, etc. Within the community; promoting positive behaviours in families through education and behaviour change communication, both through interpersonal communication and technology (integrated voice response system) are explored. Frontline health workers were trained, local leaders and volunteers were engaged, health and wellness centres within the community were set up to catalyse the community level activities. These interventions are designed to either align or complement the national guidelines [34]. Non-pharmacological interventions such as yoga and meditation that have proven effects as adjuvant methods to control certain lifestyle diseases are being explored within the community-based interventions [35]. The interventions are illustrated in Fig. 1; more details on the interventions including evaluation results will be published in the future.

The study has few limitations amidst its strengths. The study was conducted in the population catchment area of the urban PHC. Considering the wide variations in prevalence of NCDs, particularly of hypertension, a larger sample study in multiple sites would give additional insights. Though the study area is located within the city of Mysore, several parts and population segments of the study area are still in transition from rural to urban context. Hence it represents a context that is closer to urban small town or semi-urban, and not a typical metropolitan big city setting. The study focused on two common NCDs, hypertension and diabetes and hence may not necessarily be generalized to all categories of NCDs.

A challenge that most developing countries face today is the non-availability of data for planning NCD programmes comprehensively [36] This was addressed effectively in the study area. The population-wide screening of NCD and its risk factors is the first step toward planning programmes at primary level which is highlighted in the policies and guidelines [37] The current methodology that was used in the study offers several advantages. Apart from providing the population-based data, the process was time efficient, achieved near total coverage of population and helped to establish denominators of those that have disease as well as risk factors. Enabling the primary care staff to be able to update their population data on a regular basis prior to planning action, is very crucial to achieving impact at the population level. The study successfully helped program managers design interventions at different levels of care.

## Conclusion

It is important to note that the non-communicable disease programmes pertaining to diabetes and cardiovascular diseases, are not implemented comprehensively through the care-continuum, nor is backed by sound evidence for planning. This study is probably the first of its kind to have informed a comprehensive programme at primary care level. While the study focused on hypertension and diabetes, the principles and approaches are applicable to other NCDs as well. The study provided population-based data to the programmes managers, helped them to develop interventions and innovations within the existing health system and community system settings. In the next phase (2018–19), the interventions will be supported with formative research and concurrent monitoring, to enable continuous refinement of intervention strategies so as to arrive at a scalable and sustainable implementation model. The whole exercise is an excellent example of a public-private engagement in public health. The study was funded by a corporate through its corporate social responsibility (CSR) funding, the implementation was led by a civil society partner and overall leadership, approval and intervention resources were provided by the state.

## Additional files

Additional file 1: Specifiaction of diagnostics. All specifications of diagnostic equipment used for advanced test proposed are mentioned in this file. (DOCX 12 kb)

Additional file 2: Adapted WHO STEPS NCD Risk Assessment Tool. An adapted version of the WHO STEPS NCD risk assessment questionnaire. (DOCX 1526 kb)

Additional file 3: Health Seeking Behaviour and Out of Pocket expenditure assessment tool. A questionnaire for capturing health seeking and out-of-pocket health expenditure among known diabetic and hypertensive clients. (DOCX 154 kb)

Additional file 4: The Facility audit tool. A questionnaire for capturing readiness of government and private facilities. (PDF 765 kb)

Additional file 5: FGD and Semi-structure IDI guide. The Focus Group Discussion and Indepth Interview field guide. (DOCX 23 kb)

## Abbreviations

BMI: Body Mass Index
BP: Blood Pressure
CAPI: Computer-Assisted Personal Interviewing (CAPI)
CSR: Corporate Social Responsibility
CVD: Cardiovascular Diseases
ECG: Electrocardiogram
FBS: Fasting Blood Sugar
FGD: Focus Group Discussion
HbA1c: Glycated Haemoglobin A1c
IDI: Indepth Interview
KHPT: Karnataka Health Promotion Trust
LMIC: Low-and- Middle Income Countries
MBBS: Bachelor of Medicine and Bachelor of Surgery
NCD: Noncommunicable Diseases
NFHS: National Family Health Survey
PPBS: Postprandial Blood Sugar
RBS: Random Blood Sugar
SDG: Sustainable Development Goal
UPHC: Urban Primary Health Centre
WHO: World Health Organisation

## Footnotes

1 W.H.O’s South East Asian region includes the following countries: Bangladesh, Bhutan, DPR Korea, India, Indonesia, Maldives, Myanmar, Nepal, Srilanka, Thailand, Timor-Leste (source: http://www.searo.who.int/en/;

2 Constitution of India recognizes certain sections of population that are historically disadvantaged and refers to them as scheduled caste and scheduled tribes. They represent 17 and 8%, respectively of general population as per census 2011;

3 KISH table is a random number table used for selection of household member from each household for interviews. This was developed by statistician Leslie Kish in 1949, still widely used in surveys.
