# Supplementary material for: Designing a comprehensive Non-Communicable Diseases (NCD) programme for hypertension and diabetes at primary health care level: evidence and experience from urban Karnataka, South India
Source: BMC Public Health. 2019 Apr 16;19:409. doi: 10.1186/s12889-019-6735-z (PMC6469122; doi:10.1186/s12889-019-6735-z)
Supplement: Supplementary file 1 — Specifiaction of diagnostics. All specifications of diagnostic equipment used for advanced test proposed are mentioned in this file. (DOCX 12 kb) [file 12889_2019_6735_MOESM1_ESM.docx]

**Additional file 1: Specification of diagnostics used during screening**

| **S No.** | **Parameter measured** | **Equipment** | **Equipment Model** |
| --- | --- | --- | --- |
| 1 | Random Blood Sugar | Digital Glucometer | Freestyle Optium H Glucometer |
| 2 | Random Blood Sugar | Glucometer strips | Freestyle Optium H Strips |
| 3 | Random Blood Sugar | Lancets | Peerless on call- Acon Lancets |
| 4 | Blood Pressure | Digital BP Apparatus | Omron HEM 7120 BP Apparatus |
| 5 | Body Weight | Digital Weighing Machine | Omron HN 286 Digital Weighing scale |
| 6 | Height | Standard Measuring tape | Deca 201 |
