# Supplementary material for: Designing a comprehensive Non-Communicable Diseases (NCD) programme for hypertension and diabetes at primary health care level: evidence and experience from urban Karnataka, South India
Source: BMC Public Health. 2019 Apr 16;19:409. doi: 10.1186/s12889-019-6735-z (PMC6469122; doi:10.1186/s12889-019-6735-z)
Supplement: Supplementary file 2 — Adapted WHO STEPS NCD Risk Assessment Tool. An adapted version of the WHO STEPS NCD risk assessment questionnaire. (DOCX 1526 kb) [file 12889_2019_6735_MOESM2_ESM.docx]

Additional File 2: Adapted W.H.O STEPS NCD Risk Assessment Tool

Prevalence of Diabetes, Hypertension, and their associated risk factors and Health Seeking Behavior Among Adults in an Urban PHC area in Mysore City

| SECTION I: Household Identification Particulars |
| --- |
| Name of Ward_______________________________________________________________  ANM code___________________________________________________________________  Name and code of CRP: _________________________________________________  Household number…………………………………………………………………………………………………………………………………………………  Name of Household head____________________________________________________  Address of Household ____________________________________________________  ____________________________________________________  Contact Number of Household [LAND][MOBILE]  Respondent’s Line Number [**From Section II**]  Date of Interview: (**DD/MM/YYYY)** / / /  HH Result: Completed=1, Refused=2, HH Locked=3, No Competent Respondent=4, Not Dwelling Place=5  **IF RESULT NOT 1 END** |

##### SECTION II: Household Adult (>=18 years) Members

**ASK TO THE HOUSEHOLD HEAD OR ANY OTHER ADULT MEMBER AVAILABLE AT THE TIME OF VISIT ABOUT PERSONS WHO USUALLY LIVE IN THE HOUSEHOLD**

B1. Number of total household members: Male: Female:

B2. Number of total adult household members: Male: Female:

**Please give me the names of the adult persons beginning with Head of Household.**

| Line.No. | Name | What is the relationship of (NAME) to the head of the household? | Is (NAME) a male or female?  **Male=1, Female=2, TG=3** | How old is (NAME)?  **(Put 998 if Don’t know)** | What is the highest level of education completed by (NAME)?  **IF none record 00** | Can (NAME) read and write?  **Yes=1**  **No=2** | What is the current marital status of (NAME)? | ASK IF THERE ARE ANY PREGNANT WOMEN IN HOUSEHOLD  Yes=1  No=2 |
| --- | --- | --- | --- | --- | --- | --- | --- | --- |
| B3 | B4 | B5 | B6 | B7 | B8* | B9 | B10 | B11 |
| 01 |  |  |  |  |  |  |  |  |
| 02 |  |  |  |  |  |  |  |  |
| 03 |  |  |  |  |  |  |  |  |
| 04 |  |  |  |  |  |  |  |  |
| 05 |  |  |  |  |  |  |  |  |
| 06 |  |  |  |  |  |  |  |  |
| 07 |  |  |  |  |  |  |  |  |
| 08 |  |  |  |  |  |  |  |  |
| 09 |  |  |  |  |  |  |  |  |
| 11 |  |  |  |  |  |  |  |  |
| 12 |  |  |  |  |  |  |  |  |
| 13 |  |  |  |  |  |  |  |  |
| 14 |  |  |  |  |  |  |  |  |
| 15 |  |  |  |  |  |  |  |  |
| 16 |  |  |  |  |  |  |  |  |
| 17 |  |  |  |  |  |  |  |  |
| 18 |  |  |  |  |  |  |  |  |
| 19 |  |  |  |  |  |  |  |  |
| 20 |  |  |  |  |  |  |  |  |

**B5:** HH head=01, Wife/ husband=02, Son/daughter=03, Son-in-law/Daughter-in-law=04, Grandchild=05, Parent=06, Parent-in-law=07, Brother/sister=08, Brother-in-law/sister-in-law=09, Niece/nephew=10, Other relative=11, Adopted/foster child=12, Not related=13

*B8**:** IF NO FORMAL SCHOOLING PUT 00

**B10:** Currently Married=1, Widowed=2, Divorced=3, Separated=4, Deserted=5, Never Married=6

##### SECTION III: Household characteristics

**Now, I am going to ask some information about your household.**

| **No.** | **QUESTIONS AND FILTERS** | CODING CATEGORIES | **SKIP** |
| --- | --- | --- | --- |
| C1 | What is your religion? | HINDU……………………………………………….1  MUSLIM…………………………………………….2  CHRISTIAN………………………………………...3  OTHER__________________________4  (SPECIFY)________________________  NO SPECIFIC RELIGION /CAN NOT SAY_____________________________8 |  |
| C2 | What is your caste? | SCHEDULED CASTE……………………..1  SCHEDULED TRIBE………………………2  OTHER BACK WARD CASTE………………3  NONE OF THEM………………………………4  NO SPECIFIC CASTE/CANNOT SAY………………8 |  |
| C3 | Do you possess a BPL card in your family? | YES………………………………………………….......1  NO ……………………………………………………….2  DON’T KNOW ………………………………………8 |  |
| C4 | What is the type of this house?  **(OBSERVE THE ROOF, WALL, FLOOR AND CODE)** | KACCHA…………………………………………………..1  SEMI-PUCCA..………………………………………….2  PUCCA…………………………………………………….3 |  |
| C5 | **HOUSEHOLD AMENITIES**  Does your household have any of the following?  **READ LIST** | YES NO  WASHING MACHINE 1 2  MOBILE…………………………………………...1 2  TELEVISION ..1 2  REFRIGERATOR……………………………….1 2  TWO WHEELER…………………………….....1 2  THREE WHEELER………………………………1 2  FOUR WHEELER……………………………….1 2  AIR CONDITIONER/ AIR COOLER……….1 2  COMPUTER/LAPTOP…………………………1 2  INTERNET ACCESS……………………………..1 2  WATER PUMP……………………………………1 2 |  |
| C6 | What is your monthly household income from all sources?  **WRITE “000000” IF NO INCOME.** | INCOME__________  DON’T KNOW………………………………………999998  NO RESPONSE………………………………………999999 |  |
| C7 | Where do you generally get information on diabetes and hypertension?  **MULTIPLE ANSWERS POSSIBLE**  **PROBE AND MARK ALL APPLICABLE** | NOWHERE……..……………………………………………98  NEWSPAPERS / MAGAZINES………………………..01  RADIO/TV…………………………………………………....02  BILLBOARDS…………………………………………………03  BROCHURES, POSTERS, OTHER PRINTED MATERIAL…………………………………………………….04  HEALTH WORKERS……………………………………….05  NGO OUTREACH WORKERS………………………….06  COMMUNTIY MEETINGS………………………………07  VIDEO SHOW IN THE COMMUNITY………………08  TELEPHONE HELP LINE………………………………….09  ONLINE(INTERNET/SOCIAL MEDIA ETC)……………………………………………………………..10  FRIENDS/RELATIVES…………………………………….11  NO MORE….…..……………………………………………97  OTHERS______________________________88  (SPECIFY) |  |

##### **SECTION IV ONWARDS TO BE ADMINISTERED TO ALL INDIVIDUAL ADULT MEMBERS OF THE HOUSEHOLD**

##### SECTION IV: Diet

**Now, I will ask you about your daily diet pattern.**

| Line.No.  As per the Member line no. in section III | Usually how often do you eat rice  2-3 times a day=1  Once daily=2  2-3 times in a week=3  2-3 times a month=4  Occasionally=5  Never=6 | Usually how often do you eat wheat/ragi/other cereals  2-3 times a day=1  Once daily=2  2-3 times in a week=3  2-3 times a month=4  Occasionally=5  Never=6 | Usually how often do you eat pulses/legumes  2-3 times a day=1  Once daily=2  2-3 times in a week=3  2-3 times a month=4  Occasionally=5  Never=6 | Usually how often do you eat vegetables/tubers/green leafy vegetables  2-3 times a day=1  Once daily=2  2-3 times in a week=3  2-3 times a month=4  Occasionally=5  Never=6 | Usually how often do you eat processed food high in salt/oily fried foods?  More than once a day =1  Once daily=2  2-3 times in a week=3  2-3 times a month=4  Occasionally=5  Never=6 | Usually how often do you add extra salt or a salty sauce to your food right before you eat it or as you are eating it?  More than once a day =1  Once daily=2  2-3 times in a week=3  2-3 times a month=4  Occasionally=5  Never=6 | Usually how often do you eat fruits?  2-3 times a day=1  Once daily=2  2-3 times in a week=3  2-3 times a month=4  Occasionally=5  Never=6 | Usually how often do you eat meat/meat products?  2-3 times a day=1  Once daily=2  2-3 times in a week=3  2-3 times a month=4  Occasionally=5  Never=6 | How often do you eat foods high in sugar content?  2-3 times a day=1  Once daily=2  2-3 times in a week=3  2-3 times a month=4  Occasionally=5  Never=6 |
| --- | --- | --- | --- | --- | --- | --- | --- | --- | --- |
| D1 | D2 | D3 | D4 | D5 | D6 | D7 | D8 | D9 | D10 |
|  |  |  |  |  |  |  |  |  |  |
|  |  |  |  |  |  |  |  |  |  |
|  |  |  |  |  |  |  |  |  |  |
|  |  |  |  |  |  |  |  |  |  |
|  |  |  |  |  |  |  |  |  |  |
|  |  |  |  |  |  |  |  |  |  |
|  |  |  |  |  |  |  |  |  |  |
|  |  |  |  |  |  |  |  |  |  |
|  |  |  |  |  |  |  |  |  |  |
|  |  |  |  |  |  |  |  |  |  |
|  |  |  |  |  |  |  |  |  |  |
|  |  |  |  |  |  |  |  |  |  |
|  |  |  |  |  |  |  |  |  |  |
|  |  |  |  |  |  |  |  |  |  |
|  |  |  |  |  |  |  |  |  |  |
|  |  |  |  |  |  |  |  |  |  |
|  |  |  |  |  |  |  |  |  |  |
|  |  |  |  |  |  |  |  |  |  |
|  |  |  |  |  |  |  |  |  |  |

##### SECTION V: Physical Activity

**Now, I will ask you about the time you spend doing different types of physical activities.**

**Think First…**

| Line.No. **(Members line no. from SEC IV)** | What is your occupation, that is, what kind of work do you mainly do? | In a typical week how many days does your work involve vigorous-intensity activity that causes large increases in breathing or heart rate like [carrying or lifting heavy loads (20 or more kgs), cycling in gym, digging or gyming, vigorous intensity sports, construction work] for at least 10 minutes continuously?  **IF 0 GO TO E5** | How much time do you usually spend doing vigorous physical activities in a typical day?  **RECORD IN MINUTES**  **RECORD 998 IF DO NOT KNOW** | In a typical week how many days does your work involve moderate-intensity activity that causes small increases in breathing or heart rate like [brisk walking, regular cycling, carrying light (<20 kgs) loads] for at least 10 minutes continuously?  **IF 0 GO TO E7** | How much time did you usually spend doing moderate physical activities in a typical day?  **RECORD IN MINUTES**  **RECORD 998 IF DO NOT KNOW** | How much time during a typical day, do you usually spend sitting or reclining (not sleeping)?  **RECORD IN MINUTES**  **RECORD 998 IF DO NOT KNOW** |
| --- | --- | --- | --- | --- | --- | --- |
| E1 | E2 | E3 | E4 | E5 | E6 | E7 |
|  |  |  |  |  |  |  |
|  |  |  |  |  |  |  |
|  |  |  |  |  |  |  |
|  |  |  |  |  |  |  |
|  |  |  |  |  |  |  |
|  |  |  |  |  |  |  |
|  |  |  |  |  |  |  |
|  |  |  |  |  |  |  |
|  |  |  |  |  |  |  |
|  |  |  |  |  |  |  |
|  |  |  |  |  |  |  |
|  |  |  |  |  |  |  |
|  |  |  |  |  |  |  |
|  |  |  |  |  |  |  |
|  |  |  |  |  |  |  |
|  |  |  |  |  |  |  |
|  |  |  |  |  |  |  |
|  |  |  |  |  |  |  |
|  |  |  |  |  |  |  |

##### SECTION VI: Tobacco Use

**Now, I will ask you about tobacco use. I would repeat that these questions are only for the purpose of the study and I repeat your responses would not be shared with anyone else.**

| Line.No**(Members line no. from SEC IV)** | Have you ever smoked or used tobacco products in your life?  Yes=1  No=2  **IF “2”, GO TO F9** | Do you **currently** use any tobacco products, such as cigarette, beedi, chewing tobacco or snuff?  **Only Cigarette=1**  **Only Beedi=2**  **Chewing/snuff=3**  **Both Smoking & Chewing/snuff=4**  **None=5**  **IF “5” GO TO F7** | How often do you use this/these products?  **Daily=1**  **More than once in a week=2**  **Weekly once=3**  **Once in Month=4**  **Other=5** | **DO NOT ASK IF “3” in F3 & (“4” or “5”) in F4**  On average how many cigarette/beedi do you smoke daily/weekly? | **DO NOT ASK IF “1” or “2” in F3 & (“4” or “5”) in F4**  On average how many times smokeless tobacco products do you use daily/weekly? | How old were you when you first started smoking/ using tobacco products?  **RECORD AGE IN YEARS**  **98 if DO NOT REMEMBER**  **IF “5” in F3 GO TO F9** | **ONLY FOR CURRENT USERS OF TOBACCO**  In the past 12 months have you ever tried to stop smoking or using tobacco of other types?  **Yes=1**  **No=2**  **GO TO NEXT MEMBER** | **ASK ONLY TO THOSE WHO DO NOT SMOKE**  Have you ever come across, when someone in your family smoke in your home or others smoke anywhere else when you were present?  **Yes=1**  **No=2**  **IF “2” SKIP TO F11** | How frequently you come across someone who smoke when you are present  **Daily=1**  **More than once a week=2**  **Occasionally=3** | **ASK ONLY FOR THOSE CURRENTLY NOT USING ANY TOBACCO PRODUCTS**  In the past, how often did you smoke or use tobacco products?  **Daily=1**  **More than once in a week=2**  **Weekly once=3**  **Once in Month=4**  **Other=5** | Since how many months have you stopped use of tobacco?  **000 IF LESS THAN A MONTH AND 998 if DO NOT REMEMBER** |
| --- | --- | --- | --- | --- | --- | --- | --- | --- | --- | --- | --- |
| F1 | F2 | F3 | F4 | F5 | F6 | F7 | F8 | F9 | F10 | F11 | F12 |
|  |  |  |  |  |  |  |  |  |  |  |  |
|  |  |  |  |  |  |  |  |  |  |  |  |
|  |  |  |  |  |  |  |  |  |  |  |  |
|  |  |  |  |  |  |  |  |  |  |  |  |
|  |  |  |  |  |  |  |  |  |  |  |  |
|  |  |  |  |  |  |  |  |  |  |  |  |
|  |  |  |  |  |  |  |  |  |  |  |  |
|  |  |  |  |  |  |  |  |  |  |  |  |
|  |  |  |  |  |  |  |  |  |  |  |  |
|  |  |  |  |  |  |  |  |  |  |  |  |
|  |  |  |  |  |  |  |  |  |  |  |  |
|  |  |  |  |  |  |  |  |  |  |  |  |
|  |  |  |  |  |  |  |  |  |  |  |  |
|  |  |  |  |  |  |  |  |  |  |  |  |
|  |  |  |  |  |  |  |  |  |  |  |  |
|  |  |  |  |  |  |  |  |  |  |  |  |
|  |  |  |  |  |  |  |  |  |  |  |  |
|  |  |  |  |  |  |  |  |  |  |  |  |
|  |  |  |  |  |  |  |  |  |  |  |  |

##### SECTION VII: Alcohol Use

**Now, I will ask you about alcohol use. I would repeat that these questions are only for the purpose of the study and I repeat your responses would not be shared with anyone else.**

| Line.No. **(Member line no. from SEC IV)** | Have you ever consumed any alcohol such as beer, wine, spirits?  Yes=1  No=2  **IF 2 GO TO NEXT MEMBER** | Have you consumed any alcohol in the past 12 months?  Yes=1  No=2  **IF 2 GO TO G7** | What is the type of alcohol most commonly consumed by you?  Beer=1  Rum/Vodka/Whisky =2  Wine=3  Locally Prepared Alcohol= 4  Others=5 | Usually, how often do you consume alcohol in a month?  **RECORD IN DAYS**  **IF DAILY RECORD 30**  **IF NOT MONTHLY RECORD 97** | Usually, how many standard drinks containing alcohol do you have when you are drinking?  1 or 2=1  3 or 4=2  5 or 6=3  7 to 9=4  10 or more=5  Do not remember=8 | How old were you when you first started consuming alcohol?  **RECORD AGE IN YEARS**  **98 if DO NOT REMEMBER** |
| --- | --- | --- | --- | --- | --- | --- |
| G1 | G2 | G3 | G4 | G5 | G6 | G7 |
|  |  |  |  |  |  |  |
|  |  |  |  |  |  |  |
|  |  |  |  |  |  |  |
|  |  |  |  |  |  |  |
|  |  |  |  |  |  |  |
|  |  |  |  |  |  |  |
|  |  |  |  |  |  |  |
|  |  |  |  |  |  |  |
|  |  |  |  |  |  |  |
|  |  |  |  |  |  |  |
|  |  |  |  |  |  |  |
|  |  |  |  |  |  |  |
|  |  |  |  |  |  |  |
|  |  |  |  |  |  |  |
|  |  |  |  |  |  |  |
|  |  |  |  |  |  |  |
|  |  |  |  |  |  |  |
|  |  |  |  |  |  |  |
|  |  |  |  |  |  |  |

##### SECTION VIII: RBS, BP, Anthropometric Measures

**INFORM THE RESPONSDENT YOU ARE GOING TO CONDUCT THE TESTS. RECORD THE DETAILS WITH PROPER LINE NUMBER AS IN SECTION II**

**I am going to conduct the RBS, BP tests and measure your height, weight, hip, arm and waist circumference. Please relax. None of these are painful.**

| Line.No.  As per the Member line no. in section III. | Do you suffer from Diabetes or Hypertension (HIGH BP)?  Diabetes=1  Hypertension=2  Both=3  None=4  Don’t Know=8 | RBS  **IF CONSENT NOT GIVEN RECORD 998** | BP (Systolic)  **IF CONSENT NOT GIVEN RECORD 998** | BP (Diastolic)  **IF CONSENT NOT GIVEN RECORD 998** | Height  (cms)  **IF CONSENT NOT GIVEN RECORD 998** | Weight  (kgs)  **IF CONSENT NOT GIVEN RECORD 998** | Waist  (cms)  **IF CONSENT NOT GIVEN RECORD 998** | Hip  (cms)  **IF CONSENT NOT GIVEN RECORD 998** |
| --- | --- | --- | --- | --- | --- | --- | --- | --- |
| H1 | H2 | H3 | H4 | H5 | H6 | H7 | H8 | H9 |
|  |  |  |  |  |  |  |  |  |
|  |  |  |  |  |  |  |  |  |
|  |  |  |  |  |  |  |  |  |
|  |  |  |  |  |  |  |  |  |
|  |  |  |  |  |  |  |  |  |
|  |  |  |  |  |  |  |  |  |
|  |  |  |  |  |  |  |  |  |
|  |  |  |  |  |  |  |  |  |
|  |  |  |  |  |  |  |  |  |
|  |  |  |  |  |  |  |  |  |
|  |  |  |  |  |  |  |  |  |
|  |  |  |  |  |  |  |  |  |
|  |  |  |  |  |  |  |  |  |
|  |  |  |  |  |  |  |  |  |
|  |  |  |  |  |  |  |  |  |
|  |  |  |  |  |  |  |  |  |
|  |  |  |  |  |  |  |  |  |
|  |  |  |  |  |  |  |  |  |
|  |  |  |  |  |  |  |  |  |

**THANK YOU !!**
