# Supplementary material for: Designing a comprehensive Non-Communicable Diseases (NCD) programme for hypertension and diabetes at primary health care level: evidence and experience from urban Karnataka, South India
Source: BMC Public Health. 2019 Apr 16;19:409. doi: 10.1186/s12889-019-6735-z (PMC6469122; doi:10.1186/s12889-019-6735-z)
Supplement: Supplementary file 3 — Health Seeking Behaviour and Out of Pocket expenditure assessment tool. A questionnaire for capturing health seeking and out-of-pocket health expenditure among known diabetic and hypertensive clients. (DOCX 154 kb) [file 12889_2019_6735_MOESM3_ESM.docx]

##### Additional File 3: Assessment of Health Seeking Behaviour and Out of Pocket Expenses (Only for Diabetic Or Hypertensive clients)

**Now, I will ask you about where you take your treatment from and the cost of medication.**

| Line.No.  Of Members Diagnosed with Diabetes or Hypertension | **STATUS OF DIABETES AND BP FROM SECTION VIII**  Knew, Diabetic=1  Knew,  Hypertensive=2  Knew Both=3  Identified Now, ONLY Diabetic=4  Identified Now, ONLY Hypertensive=5  Identified Now, Both=6  Knew Diabetic, identified now hypertensive=7  Knew hypertensive, identified now diabetic=8 | From this list where would/do you visit (often) for treatment of your diabetes/hypertension or both?  Government hospital (DH/TH/ CHC)=1  UPHC=2  Private Hospital/Clinic=3  NGO Hospital/Clinic=4  Private Pharmacy=5  Others=6  No Where=7  Do Not Know=8  **IF “7” or “8“ GO TO I6** | Name of Preferred Facility and Provider  Address  IF “4” or “5” or “6” in I2 END | **IF I2 is “4/5/6” THEN GO TO NEXT**  How many times did you visit the facility for treatment in last 12 months?  **RECORD NUMBER** | **IF I2 is “4/5/6” THEN GO TO NEXT**  How much did you spend from your own pocket towards medicines, consultation, investigations for diabetes or hypertension in the **last six months**?  **RECORD 998 IF DO NOT KNOW**  **M: Med**  **I: INV**  **C: CON** | How did you pay for the medicines, investigations and consultancy for your diabetes or hypertension or both?  **No Medication/treatment=0**  Free of Cost form govt. hospital=1  Pay out of pocket=2  Partially covered by insurance scheme=3  Completely covered by insurance scheme=4  **M: Med**  **I: INV**  **C: CON** | **IF I7 “0” THEN GO TO I13 ELSE**  **ASK ONLY IF I2 is “1” or “3” or “7”**  Do you have your medicines for diabetes at home today?  **IF Yes, ASK TO SHOW THE MEDICINES AND RECORD ACCORDINGLY**  **Yes, Shown=1**  **Yes, Not Shown=2**  **No=3** | **ASK ONLY IF I2 is “2” or “3” or “8”**  Do you have your medicines for hypertension at home today?  **IF Yes, ASK TO SHOW THE MEDICINES AND RECORD ACCORDINGLY**  **Yes, Shown=1**  **Yes, Not Shown=2**  **No=3** | **ASK FOR THE PRESCRIPTION**  IF PRESCRIPTION AVAILABLE- RECORD Names of medicines  Consumed  **(Only those for diabetes or hypertension)** | Please mention the number of days for which medicines are obtained by you at one time usually? | Have you been able to take medicines as directed?  Yes, fully able to=1  Partially able to=2  Not at all=3  **IF 1 OR 2 Go TO NEXT MEMBER** | ONLY FOR THOSE NOT TAKING TREATMENT/MEDICATIONS EVEN AFTER KNOWING THEIR STATUS  What is/are the reason/s for not fully able to take medicines as directed OR not taking treatment?  **MULTIPLE ANSWERS POSSIBLE PROBE AND MARK ALL APPLICABLE**  **Could not travel to facility=A**  **Medicine not available at facility/shop= B**  **No money to buy medicines/treatment=C**  **Side effects of medicines = D**  **Forgot to take medicines as per prescription= E**  **Do not know where to go= F**  **Do not think that this needs treatment= G**  **Self-test at home=H**  **Other=X** |
| --- | --- | --- | --- | --- | --- | --- | --- | --- | --- | --- | --- | --- |
| I1 | I2 | I3 | I4 | I5 | I6 | I7 | I8 | I9 | I10 | I11 | I12 | I13 |
|  |  |  |  |  | A)  B)  C) | A)  B)  C) |  |  |  |  |  |  |
|  |  |  |  |  | A)  B)  C) | A)  B)  C) |  |  |  |  |  |  |
|  |  |  |  |  | A)  B)  C) | A)  B)  C) |  |  |  |  |  |  |
|  |  |  |  |  | A)  B)  C) | A)  B)  C) |  |  |  |  |  |  |
|  |  |  |  |  | A)  B)  C) | A)  B)  C) |  |  |  |  |  |  |
|  |  |  |  |  | A)  B)  C) | A)  B)  C) |  |  |  |  |  |  |
