# Supplementary material for: Designing a comprehensive Non-Communicable Diseases (NCD) programme for hypertension and diabetes at primary health care level: evidence and experience from urban Karnataka, South India
Source: BMC Public Health. 2019 Apr 16;19:409. doi: 10.1186/s12889-019-6735-z (PMC6469122; doi:10.1186/s12889-019-6735-z)
Supplement: Supplementary file 4 — The Facility audit tool. A questionnaire for capturing readiness of government and private facilities. (PDF 765 kb) [file 12889_2019_6735_MOESM4_ESM.pdf]

Strethening continuum of care for select NCDs in an urban PHC area of Mysuru, Karnataka

Facility Checklist

IDENTIFICATION	Code
**Ward no**	☐
**ANM area**	☐
**FACILITY NAME:**	
**FACILITY TYPE**<br><br>1. TH<br>**2.** UCHC<br>**3.** UPHC<br>**4.** PRIVATE Hospital<br>**5.** PRIVATE Clinic<br>**6.** AYUSH Hospital<br>**7.** AYUSH Clinic<br>**8.** NGO Hospital<br>**9.** NGO Clinic	☐
**DATE OF ASSESSMENT:** DAY ☐☐ MONTH ☐☐ YEAR ☐☐☐☐	
**NAME OF INVESTIGATOR**..	☐☐

Human Workforce

Positions	Are these Staff available?<br><br>Available 7 days a week=1<br>Partial in the week=2<br>Not Available=3	If Available Full time or Partially, record the number of staff
Specialist – Endocrinologist/diabetologist/General Physicial MD		
Specialist- Cardiologist		
Medical officer-MBBS		
Medical officer-AYUSH		
Pharmacist		
Laboratory Technician		
Counsellor (providing tips on life style modification and health education)		
Staff Nurse		
Data Entry Operator (DEO)		
Group D		

General Service Readiness

**Basic Equipment**	**Yes= 1 No=2**
Adult Weight Scale	
Blood Pressure Apparatus	
Stethoscope	
Stature / Stadio Meter *(Height measurement)*	
**Diagnostic Services**	
Blood glucose- FBS, PPBS	
HbA1C	
Lipid Profile	
ECG	
ECHO	
**Diagnostic Capacities to identify complications**	
Diabetic Retinopathy-Retinopathy screening through Ophthalmoscopy	
Diabetic Nephropathy-Kidney Function Test *(B.Urea and S.Creatinine)*	
Foot examination to assess diabetic neuropathy	
**Medicines**	
Oral Anti-Diabetic *(T.Metformin 500mg, T.Gilibenclamide 5 mg, T.Glimipride 1 mg etc)*	
Oral Anti-Hypertensive *(T.HCTZ 12.5 mg, T.Enalapril 5 mg, T.Amlodipine 5mg, T.Atenolol 25 mg, T. Temisartan 40 mg etc)*	
Insulin- Injection/ Premix	
Anti dyslipidemia drugs (T. Atorvastatin 10 mg etc)	
**Registers**	
NCD screening register / follow up register	
Laboratory register	
