# Supplementary material for: Designing a comprehensive Non-Communicable Diseases (NCD) programme for hypertension and diabetes at primary health care level: evidence and experience from urban Karnataka, South India
Source: BMC Public Health. 2019 Apr 16;19:409. doi: 10.1186/s12889-019-6735-z (PMC6469122; doi:10.1186/s12889-019-6735-z)
Supplement: Supplementary file 5 — FGD and Semi-structure IDI guide. The Focus Group Discussion and Indepth Interview field guide. (DOCX 23 kb) [file 12889_2019_6735_MOESM5_ESM.docx]

**Additional File 5: Qualitative FGD & In-depth Interview Guide**

**Objective:**

To understand the adult’s views, perspective and experiences on diabetes and hypertension treatment access, adherence and life style modifications in an urban primary health centre context in Mysore

**Research questions:**

1. What are the motivators and barriers for adults to:
   1. visit facility for confirmatory test and initiation of treatment?
   2. adhere to the medications?
   3. accept for lifestyle modification?
2. **Socio-demographic details :**
3. Age :_________________
4. Gender : _________________
5. Qualification : _________________
6. Occupation : _________________
7. Marital status : _________________
8. Income (monthly) : _________________
9. **General health seeking behaviour:**
10. Generally, where does people go for treatment? (Probe for where do they prefer to go first for treatment). Where do you prefers to go? Did you face any difficulties there? How was your experience? Please describe.
11. How does the family support you for seeking treatment? Who takes decision whether you should take treatment or not?
12. Do you use any other way to heal the disease? (Probe for the home remedies, natural healers for any specific illness) Please explain.
13. What is your opinion regarding English medicine and home remedies? Which one is most effective? Why?
14. **Knowledge, Attitude and Practice on Hypertension and Diabetes**
15. In your opinion what is diabetes and hypertension? (Probe for what they know about diabetes and hypertension, if any misconception explores)?
16. What could be the reasons for one to get hypertension and diabetes?
17. How does family/friends/ colleague/ community see a person with diabetes and hypertension? (probe for the stigma, discrimination, stereotypes) Why? What other challenges they face?
18. When did you come to know that you have hypertension and diabetes? And where it was tested? Probe for whether they gone for testing voluntarily or someone referred. Explore the testing process he or she has undergone?
19. What challenges did you faced once you know your health status? Probe for challenges related to treatment, discrimination/ stereotypes from family / friends/ colleague/ community?
20. Do you ever think that you could get better without any professionals help/ moving to hospital for treatment? Why? Or can you do it on your own or do you feel you need any professional help? If yes, what kind of help you expect? Why?
21. Did you ever felt that you have benefited from undergone the treatment/screening? How?
22. Do you think you will be able to resolve your present health problems that brought you here today? If yes, how? If no, why?
23. **Reasons for not accessing the treatment**
24. What could be reason for some people to access and others are not accessing the treatment to diabetes and hypertension? (probe for the motivational factors for some to access the treatment and barriers for others to not accessing the treatment) Please give with enough details.
25. What kind of test was performed to you? Where is the test performed? (probe about their prefer time and place for treatment) How would you describe your experience with this testing method? Did you face any problems in testing method? Please describe.
26. In your experience what could be done for people to access the treatment soon after the diagnosis?
27. **Adherence challenges for treatment**
28. Where do people prefer to go for diabetes and hypertension treatment? (probe for govt/private hospital/ natural healers)Why? Are you comfortable speaking with the health professionals? Did you face any challenges in speaking with health professionals?
29. Did you think that some people are regular to the treatment and what could be the reasons? Some others are not adherent to the treatment, what could be the reasons?
30. Does people suffering from diabetes and hypertension have any expectations regarding the treatment? If yes please explain?
31. How do you want your health care provider to perceive you?
32. Have you ever had any negative experiences with a treatment/ screening? Explain.
33. **Life style modification**
34. In your opinion, how do the diabetic and hypertension people should lead their life? What kind of lifestyle modification required?
35. Do you think consuming alcohol and tobacco can worsen the disease? How?
36. Do you have any idea what changes should be done in your/ community lifestyle to prevent diabetes? Explain in detail
37. Do you meditate? What else do you do to help your own wellness?

# Thank you for taking your time to participate in this interview!
